# Phenyl *N*-cyclo­hexyl­carbamate

**DOI:** 10.1107/S1600536809050806

**Published:** 2009-12-04

**Authors:** Durre Shahwar, M. Nawaz Tahir, Naeem Ahmad, Sami Ullah, Muhammad Akmal Khan

**Affiliations:** a Department of Chemistry, Government College University, Lahore, Pakistan; b Department of Physics, University of Sargodha, Sargodha, Pakistan

## Abstract

In the title compound, C_13_H_17_NO_2_, the dihedral angle between the benzene ring and the basal plane of the cyclo­hexyl ring is 49.55 (8)°. In the crystal, mol­ecules are linked by N—H⋯O hydrogen bonds, forming chains propagating in [010].

## Related literature

For related structures, see: Shahwar *et al.* (2009*a* ▶,*b* ▶, 2010 ▶).
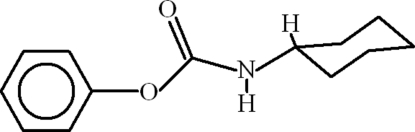


## Experimental

### 

#### Crystal data

C_13_H_17_NO_2_
*M* *_r_* = 219.28
Monoclinic, 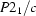

*a* = 11.4724 (11) Å
*b* = 9.3554 (8) Å
*c* = 11.5212 (10) Å
β = 92.380 (5)°
*V* = 1235.49 (19) Å^3^
*Z* = 4
Mo *K*α radiation
μ = 0.08 mm^−1^
*T* = 296 K
0.28 × 0.11 × 0.09 mm

#### Data collection

Bruker Kappa APEXII CCD diffractometer
Absorption correction: multi-scan (*SADABS*; Bruker, 2005 ▶) *T*_min_ = 0.987, *T*_max_ = 0.993
10855 measured reflections
2265 independent reflections
1207 reflections with *I* > 2σ(*I*)
*R*_int_ = 0.043

#### Refinement

*R*[*F* ^2^ > 2σ(*F* ^2^)] = 0.046
*wR*(*F* ^2^) = 0.127
*S* = 0.99
2265 reflections
145 parameters
H atoms treated by a mixture of independent and constrained refinement
Δρ_max_ = 0.12 e Å^−3^
Δρ_min_ = −0.16 e Å^−3^

### 

Data collection: *APEX2* (Bruker, 2007 ▶); cell refinement: *SAINT* (Bruker, 2007 ▶); data reduction: *SAINT*; program(s) used to solve structure: *SHELXS97* (Sheldrick, 2008 ▶); program(s) used to refine structure: *SHELXL97* (Sheldrick, 2008 ▶); molecular graphics: *ORTEP-3* (Farrugia, 1997 ▶) and *PLATON* (Spek, 2009 ▶); software used to prepare material for publication: *WinGX* (Farrugia, 1999 ▶) and *PLATON*.

## Supplementary Material

Crystal structure: contains datablocks global, I. DOI: 10.1107/S1600536809050806/hb5247sup1.cif

Structure factors: contains datablocks I. DOI: 10.1107/S1600536809050806/hb5247Isup2.hkl

Additional supplementary materials:  crystallographic information; 3D view; checkCIF report

## Figures and Tables

**Table 1:** Hydrogen-bond geometry (Å, °)

*D*—H⋯*A*	*D*—H	H⋯*A*	*D*⋯*A*	*D*—H⋯*A*
N1—H1*N*⋯O2^i^	0.849 (19)	2.018 (19)	2.865 (2)	175 (2)

Symmetry code: (i) 
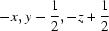
.

**Fig. 1.**
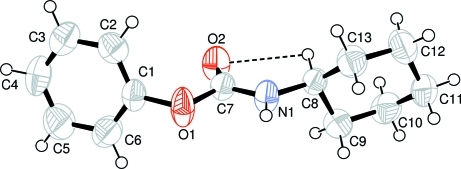
View of (I) with displacement ellipsoids drawn at the 50% probability level.

**Fig. 2.**
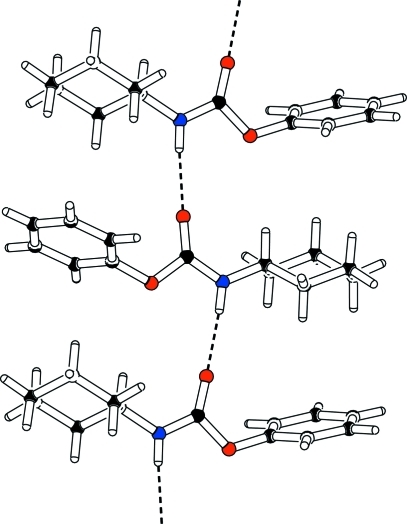
The partial packing of (I), which shows that molecules form infinite chains.

## supplementary crystallographic information

### Comment

The crystal structures of (II) phenyl piperidine-1-carboxylate (Shahwar *et al.*, 2010), (III) phenyl *N*-(2-methylphenyl)carbamate (Shahwar *et al.*, 2009*a*) and (IV) phenyl *N*-phenylcarbamate (Shahwar *et al.*, 2009*b*) have been reported by us. In continuation to synthesize various carbamates for the study of biological activities, the title compound (I, Fig. 1) is being reported.

In (I), the benzene ring A (C1—C6) is of course planar. The central carbamate group B (O1/O2/C7/N1) and the basal plane C (C9/C10/C12/C13) of cyclohexyl are also planar with maximum r. m. s. deviations of 0.002 and 0.005 Å respectively, from the respective mean square planes. The dihedral angles between A/B, B/C and A/C are 76.26 (8)°, 70.99 (9)° and 52.17 (7)° respectively. The cyclohexyl ring is in the chair conformation with the apical atoms C8 and C11 are at a distance of 0.652 (3) and -0.668 (4) Å respectively, from the basal plane (C9/C10/C12/C13). The molecules are stabilized in the form of polymeric chains (Table 1, Fig. 2).

### Experimental

Cyclohexylamine (0.01 *M*, 1.15 ml) and triethylamine (0.012 *M*, 1.66 ml) were added to 20 ml dichloromethane in a 50 ml round bottom flask equipped with magnetic stirrer. Phenyl chloroformate (0.01 *M*, 1.26 ml) was added drop wise with continuous stirring of the contents of the flask. After complete addition the stirring was continued for 30 minutes. Extra dichloromethane was evaporated and then resulting solid was washed with 1*M* HCl and filtered to get pure product. Recrystallization of the crude product with ethyl acetate affoarded colourless needles of (I).

### Refinement

The coordinates of H1N were located in a difference map and refined. The other H-atoms were positioned geometrically (C–H = 0.93–0.97 Å) and refined as riding with *U*_iso_(H) = 1.2*U*_eq_(Carrier).

### Crystal data

**Table:**

C_13_H_17_NO_2_	*F*(000) = 472
*M**_r_* = 219.28	*D*_x_ = 1.179 Mg m^−^^3^
Monoclinic, *P*2_1_/*c*	Mo *K*α radiation, λ = 0.71073 Å
Hall symbol: -P 2ybc	Cell parameters from 2265 reflections
*a* = 11.4724 (11) Å	θ = 2.8–25.4°
*b* = 9.3554 (8) Å	µ = 0.08 mm^−^^1^
*c* = 11.5212 (10) Å	*T* = 296 K
β = 92.380 (5)°	Needle, colourless
*V* = 1235.49 (19) Å^3^	0.28 × 0.11 × 0.09 mm
*Z* = 4	

### Data collection

**Table:**

Bruker Kappa APEXII CCD diffractometer	2265 independent reflections
Radiation source: fine-focus sealed tube	1207 reflections with *I* > 2σ(*I*)
graphite	*R*_int_ = 0.043
Detector resolution: 7.90 pixels mm^-1^	θ_max_ = 25.4°, θ_min_ = 2.8°
ω scans	*h* = −13→13
Absorption correction: multi-scan (*SADABS*; Bruker, 2005)	*k* = −11→11
*T*_min_ = 0.987, *T*_max_ = 0.993	*l* = −13→13
10855 measured reflections	

### Refinement

**Table:**

Refinement on *F*^2^	Primary atom site location: structure-invariant direct methods
Least-squares matrix: full	Secondary atom site location: difference Fourier map
*R*[*F*^2^ > 2σ(*F*^2^)] = 0.046	Hydrogen site location: inferred from neighbouring sites
*wR*(*F*^2^) = 0.127	H atoms treated by a mixture of independent and constrained refinement
*S* = 0.99	*w* = 1/[σ^2^(*F*_o_^2^) + (0.0463*P*)^2^ + 0.1974*P*] where *P* = (*F*_o_^2^ + 2*F*_c_^2^)/3
2265 reflections	(Δ/σ)_max_ < 0.001
145 parameters	Δρ_max_ = 0.12 e Å^−^^3^
0 restraints	Δρ_min_ = −0.16 e Å^−^^3^

### Special details

**Table:**

Geometry. Bond distances, angles *etc*. have been calculated using the rounded fractional coordinates. All su's are estimated from the variances of the (full) variance-covariance matrix. The cell e.s.d.'s are taken into account in the estimation of distances, angles and torsion angles
Refinement. Refinement of *F*^2^ against ALL reflections. The weighted *R*-factor *wR* and goodness of fit *S* are based on *F*^2^, conventional *R*-factors *R* are based on *F*, with *F* set to zero for negative *F*^2^. The threshold expression of *F*^2^ > σ(*F*^2^) is used only for calculating *R*-factors(gt) *etc*. and is not relevant to the choice of reflections for refinement. *R*-factors based on *F*^2^ are statistically about twice as large as those based on *F*, and *R*- factors based on ALL data will be even larger.

### Fractional atomic coordinates and isotropic or equivalent isotropic displacement parameters (Å^2^)

**Table:**

	*x*	*y*	*z*	*U*_iso_*/*U*_eq_	
O1	−0.09944 (15)	0.52058 (14)	0.31355 (14)	0.0774 (7)	
O2	−0.03969 (14)	0.74215 (15)	0.26826 (13)	0.0704 (6)	
N1	0.06141 (17)	0.54662 (18)	0.21748 (16)	0.0592 (7)	
C1	−0.1882 (2)	0.5766 (2)	0.3782 (2)	0.0562 (9)	
C2	−0.16451 (18)	0.62858 (16)	0.48728 (15)	0.0657 (10)	
C3	−0.25438 (18)	0.67436 (16)	0.55214 (15)	0.0756 (10)	
C4	−0.3657 (3)	0.6653 (3)	0.5093 (3)	0.0874 (12)	
C5	−0.3892 (3)	0.6127 (3)	0.4005 (3)	0.0941 (12)	
C6	−0.2992 (3)	0.5681 (3)	0.3342 (2)	0.0769 (11)	
C7	−0.0243 (2)	0.6158 (2)	0.26526 (17)	0.0513 (8)	
C8	0.15329 (19)	0.6175 (2)	0.15547 (17)	0.0503 (8)	
C9	0.1297 (2)	0.6138 (2)	0.02537 (18)	0.0640 (9)	
C10	0.2269 (2)	0.6843 (3)	−0.03874 (19)	0.0759 (10)	
C11	0.3421 (2)	0.6149 (3)	−0.0078 (2)	0.0790 (11)	
C12	0.3672 (2)	0.6198 (3)	0.1221 (2)	0.0861 (11)	
C13	0.2694 (2)	0.5510 (3)	0.18732 (19)	0.0687 (10)	
H1N	0.0588 (19)	0.456 (2)	0.2198 (17)	0.0710*	
H2	−0.08804	0.63285	0.51718	0.0788*	
H3	−0.23893	0.71187	0.62593	0.0905*	
H4	−0.42659	0.69512	0.55435	0.1045*	
H5	−0.46588	0.60696	0.37127	0.1126*	
H6	−0.31450	0.53240	0.25975	0.0923*	
H8	0.15598	0.71776	0.17996	0.0604*	
H9A	0.05675	0.66261	0.00635	0.0768*	
H9B	0.12155	0.51529	0.00004	0.0768*	
H10A	0.21067	0.67673	−0.12180	0.0910*	
H10B	0.23069	0.78500	−0.01876	0.0910*	
H11A	0.40367	0.66398	−0.04704	0.0947*	
H11B	0.34065	0.51622	−0.03374	0.0947*	
H12A	0.37630	0.71850	0.14666	0.1033*	
H12B	0.43979	0.57023	0.14083	0.1033*	
H13A	0.26646	0.44966	0.16960	0.0825*	
H13B	0.28555	0.56132	0.27021	0.0825*	

### Atomic displacement parameters (Å^2^)

**Table:**

	*U*^11^	*U*^22^	*U*^33^	*U*^12^	*U*^13^	*U*^23^
O1	0.0806 (13)	0.0403 (9)	0.1156 (13)	−0.0035 (8)	0.0577 (11)	−0.0012 (8)
O2	0.0816 (13)	0.0329 (8)	0.0991 (12)	0.0038 (8)	0.0325 (9)	0.0029 (8)
N1	0.0672 (14)	0.0331 (9)	0.0796 (14)	−0.0014 (10)	0.0321 (11)	0.0008 (9)
C1	0.0607 (18)	0.0409 (12)	0.0687 (16)	0.0029 (11)	0.0232 (14)	0.0050 (11)
C2	0.0613 (18)	0.0572 (14)	0.0786 (18)	−0.0003 (12)	0.0025 (14)	0.0031 (13)
C3	0.094 (2)	0.0744 (17)	0.0594 (16)	0.0046 (16)	0.0156 (16)	0.0005 (12)
C4	0.077 (2)	0.102 (2)	0.086 (2)	0.0202 (17)	0.0361 (18)	0.0012 (16)
C5	0.056 (2)	0.127 (2)	0.099 (2)	0.0117 (16)	0.0010 (17)	−0.0054 (19)
C6	0.078 (2)	0.0915 (19)	0.0615 (17)	0.0040 (15)	0.0065 (16)	−0.0072 (13)
C7	0.0600 (16)	0.0359 (12)	0.0593 (13)	−0.0039 (11)	0.0165 (11)	0.0001 (10)
C8	0.0569 (16)	0.0398 (11)	0.0553 (14)	−0.0073 (10)	0.0152 (11)	−0.0018 (9)
C9	0.0624 (18)	0.0666 (15)	0.0628 (15)	−0.0008 (12)	0.0012 (13)	0.0054 (11)
C10	0.093 (2)	0.0819 (17)	0.0537 (15)	−0.0121 (16)	0.0139 (15)	0.0079 (12)
C11	0.072 (2)	0.097 (2)	0.0701 (18)	−0.0193 (15)	0.0284 (15)	−0.0077 (14)
C12	0.0556 (19)	0.124 (2)	0.0791 (19)	−0.0113 (16)	0.0093 (14)	0.0037 (16)
C13	0.0644 (19)	0.0845 (17)	0.0572 (15)	0.0016 (13)	0.0025 (13)	0.0076 (12)

### Geometric parameters (Å, °)

**Table:**

O1—C1	1.389 (3)	C12—C13	1.519 (3)
O1—C7	1.373 (3)	C2—H2	0.9300
O2—C7	1.196 (2)	C3—H3	0.9300
N1—C7	1.317 (3)	C4—H4	0.9300
N1—C8	1.457 (3)	C5—H5	0.9300
N1—H1N	0.849 (19)	C6—H6	0.9300
C1—C2	1.364 (3)	C8—H8	0.9800
C1—C6	1.353 (4)	C9—H9A	0.9700
C2—C3	1.367 (3)	C9—H9B	0.9700
C3—C4	1.353 (4)	C10—H10A	0.9700
C4—C5	1.363 (5)	C10—H10B	0.9700
C5—C6	1.375 (5)	C11—H11A	0.9700
C8—C13	1.502 (3)	C11—H11B	0.9700
C8—C9	1.512 (3)	C12—H12A	0.9700
C9—C10	1.514 (3)	C12—H12B	0.9700
C10—C11	1.502 (3)	C13—H13A	0.9700
C11—C12	1.513 (3)	C13—H13B	0.9700
			
C1—O1—C7	117.32 (15)	C1—C6—H6	120.00
C7—N1—C8	123.31 (17)	C5—C6—H6	120.00
C7—N1—H1N	116.7 (15)	N1—C8—H8	108.00
C8—N1—H1N	119.8 (14)	C9—C8—H8	108.00
O1—C1—C2	120.4 (2)	C13—C8—H8	108.00
O1—C1—C6	118.5 (2)	C8—C9—H9A	109.00
C2—C1—C6	121.0 (2)	C8—C9—H9B	109.00
C1—C2—C3	119.29 (19)	C10—C9—H9A	109.00
C2—C3—C4	120.2 (2)	C10—C9—H9B	109.00
C3—C4—C5	120.4 (3)	H9A—C9—H9B	108.00
C4—C5—C6	119.8 (3)	C9—C10—H10A	110.00
C1—C6—C5	119.4 (2)	C9—C10—H10B	110.00
O1—C7—O2	122.3 (2)	C11—C10—H10A	109.00
O1—C7—N1	110.04 (16)	C11—C10—H10B	109.00
O2—C7—N1	127.7 (2)	H10A—C10—H10B	108.00
N1—C8—C9	111.85 (17)	C10—C11—H11A	110.00
C9—C8—C13	110.68 (17)	C10—C11—H11B	110.00
N1—C8—C13	110.12 (17)	C12—C11—H11A	110.00
C8—C9—C10	111.62 (18)	C12—C11—H11B	109.00
C9—C10—C11	110.8 (2)	H11A—C11—H11B	108.00
C10—C11—C12	110.56 (19)	C11—C12—H12A	109.00
C11—C12—C13	111.24 (19)	C11—C12—H12B	109.00
C8—C13—C12	111.7 (2)	C13—C12—H12A	109.00
C1—C2—H2	120.00	C13—C12—H12B	109.00
C3—C2—H2	120.00	H12A—C12—H12B	108.00
C2—C3—H3	120.00	C8—C13—H13A	109.00
C4—C3—H3	120.00	C8—C13—H13B	109.00
C3—C4—H4	120.00	C12—C13—H13A	109.00
C5—C4—H4	120.00	C12—C13—H13B	109.00
C4—C5—H5	120.00	H13A—C13—H13B	108.00
C6—C5—H5	120.00		
			
C7—O1—C1—C2	−75.3 (2)	C1—C2—C3—C4	1.4 (3)
C7—O1—C1—C6	109.8 (2)	C2—C3—C4—C5	−1.1 (4)
C1—O1—C7—O2	−7.1 (3)	C3—C4—C5—C6	0.3 (4)
C1—O1—C7—N1	173.43 (18)	C4—C5—C6—C1	0.2 (4)
C8—N1—C7—O1	177.79 (18)	N1—C8—C9—C10	−178.57 (18)
C8—N1—C7—O2	−1.7 (4)	C13—C8—C9—C10	−55.4 (2)
C7—N1—C8—C9	−98.5 (2)	N1—C8—C13—C12	178.63 (18)
C7—N1—C8—C13	138.0 (2)	C9—C8—C13—C12	54.5 (2)
O1—C1—C2—C3	−175.67 (16)	C8—C9—C10—C11	56.9 (2)
C6—C1—C2—C3	−0.9 (3)	C9—C10—C11—C12	−56.8 (3)
O1—C1—C6—C5	175.0 (2)	C10—C11—C12—C13	56.1 (3)
C2—C1—C6—C5	0.1 (4)	C11—C12—C13—C8	−55.3 (3)

### Hydrogen-bond geometry (Å, °)

**Table:**

*D*—H···*A*	*D*—H	H···*A*	*D*···*A*	*D*—H···*A*
N1—H1N···O2^i^	0.849 (19)	2.018 (19)	2.865 (2)	175 (2)

Symmetry codes: (i) −*x*, *y*−1/2, −*z*+1/2.
